# Detection of Monkeypox Virus according to The Collection Site of Samples from Confirmed Cases: A Systematic Review

**DOI:** 10.3390/tropicalmed8010004

**Published:** 2022-12-22

**Authors:** Darwin A. León-Figueroa, Joshuan J. Barboza, Hortencia M. Saldaña-Cumpa, Emilly Moreno-Ramos, D. Katterine Bonilla-Aldana, Mario J. Valladares-Garrido, Ranjit Sah, Alfonso J. Rodriguez-Morales

**Affiliations:** 1 Facultad de Medicina Humana, Universidad de San Martín de Porres, Chiclayo 15011, Peru; 2 Centro de Investigación en Atención Primaria en Salud, Universidad Peruana Cayetano Heredia, Lima 15102, Peru; 3 Vicerrectorado de Investigación, Universidad Norbert Wiener, Lima 15046, Peru; 4 Universidad San Ignacio de Loyola, Lima 13008, Peru; 5 Research Unit, Universidad Continental, Huancayo 12000, Peru; 6 Oficina de Epidemiología, Hospital Regional Lambayeque, Chiclayo 14012, Peru; 7 Facultad de Medicina, Universidad Cesar Vallejo, Piura 20002, Peru; 8 Institute of Medicine, Tribhuvan University Teaching Hospital, Kathmandu 44600, Nepal; 9 Dr. D.Y Patil Medical College, Hospital and Research Center, Dr. D.Y. Patil Vidyapeeth, Pune 411018, Maharashtra, India; 10 Grupo de Investigación Biomedicina, Faculty of Medicine, Fundacion Universitaria Autonoma de las Americas, Pereira 660001, Risaralda, Colombia; 11 Latin American Network of Monkeypox Virus Research (LAMOVI), Pereira 660003, Risaralda, Colombia; 12 Master of Clinical Epidemiology and Biostatistics, Universidad Cientifica del Sur, Lima 15067, Peru; 13 Gilbert and Rose-Marie Chagoury School of Medicine, Lebanese American University, Beirut 1102, Lebanon

**Keywords:** monkeypox, samples, orthopoxvirus, monkeypox virus, systematic review

## Abstract

Due to the rapid evolution of the monkeypox virus, the means by which the monkeypox virus is spread is subject to change. Therefore, the present study aims to analyze the detection of the monkeypox virus according to the collection site of samples from confirmed monkeypox cases. A systematic literature review was performed using PubMed, Scopus, Web of Science, and Embase databases until 5 October 2022. A total of 1022 articles were retrieved using the search strategy. After removing duplicates (*n* = 566) and examining by title, abstract, and full text, 65 studies reporting monkeypox case reports were included with a detailed description of risk factors, sexually transmitted infections (STIs), site of monkeypox virus-positive specimens, location of skin lesions, and diagnostic test. A total of 4537 confirmed monkeypox cases have been reported, of which 98.72% of the cases were male with a mean age of 36 years, 95.72% had a sexual behavior of being men who have sex with men, and 28.1% had human immunodeficiency virus (HIV). The most frequent locations of lesions in patients diagnosed with monkeypox were: 42.85% on the genitalia and 37.1% in the perianal region. All confirmed monkeypox cases were diagnosed by reverse transcriptase polymerase chain reaction (RT-PCR), and the most frequent locations of samples collected for diagnosis that tested positive for monkeypox virus were: 91.85% from skin lesions, 20.81% from the oropharynx, 3.19% from blood, and 2.43% from seminal fluid. The disease course of the cases with monkeypox was asynchronous, with no severe complications, and most patients did not report specific treatment but simply followed a symptomatic treatment.

## 1. Introduction

The global resurgence of confirmed cases of monkeypox (MPX) [1] and the rapid spread of cases in different endemic and non-endemic countries of the world have led to a public health emergency of international concern [2,3]. As of 10 December 2022, 82,474 confirmed cases of monkeypox have been reported in 110 countries worldwide [4].

Monkeypox is a reemerging zoonotic viral disease caused by the monkeypox virus (MPXV) [5]. MPXV is a double-stranded DNA virus of the genus Orthopoxvirus of the family Poxviridae known for more than half a century [6] but geographically endemic to Central and West Africa [7,8]. MPXV has two distinct clades: the Central Basin clade and the West African clade [9]. The West African clade exhibits less virulence with a mortality rate of less than 1% [10]. On the other hand, the Central Basin clade (Central African clade) is more lethal, with a mortality rate of up to 10% in unvaccinated children [11]. This is because the two clades are genetically distinct [10]. The Central African clade is reported more frequently than the West African clade and has documented cases of person-to-person transmission, whereas the West African clade does not [12].

Monkeypox is transmitted to humans through close contact with an infected animal or person through lesions, body fluids, respiratory droplets, or virus-contaminated material [13]. The disease has an incubation period of 5 to 21 days [14] with nonspecific clinical manifestations such as fever, lymphadenopathy, headache, malaise, and general lesions [15,16].

Polymerase chain reaction (PCR) using swabs of skin lesions (vesicles, ulcers, and scabs) recommended for confirming infection in symptomatic individuals is used to detect MPXV [17]. In addition, MPXV DNA has been detected by PCR in a wide variety of samples such as throat, nasopharynx, blood, urine, saliva, and semen [18].

It is possible for a person to be infected with MPXV and not show any symptoms [19]. In asymptomatic individuals, MPXV has been found in urethral [20] and anal swabs [20,21,22], according to recent research. In addition, persons with asymptomatic MPX can likely transmit the virus.

Currently, knowledge about the means by which MPXV is spread is rapidly evolving. The rapid increase in MPX cases in non-endemic areas has challenged clinical laboratories, testing has been limited, and results have been delayed [23]. The excretion and transmission of MPXV are poorly understood, and relevant data to support clinical management and public health response are lacking [24].

Therefore, the present study aims to analyze the detection of the monkeypox virus according to the collection site of samples from confirmed monkeypox cases.

## 2. Materials and Methods

### 2.1. Protocol and Registration

This protocol follows the recommendations established by the Preferred Reporting Items for Systematic Reviews and Meta-Analyses (PRISMA) statement, and it has been reported in the International Prospective Register of Systematic Reviews (PROSPERO) database (CRD42022368207).

### 2.2. Eligibility Criteria

Published peer-reviewed articles with study designs of case reports, case series, and observational studies (nonrandomized cohort and intervention studies) were included to investigate monkeypox virus detection by the location of specimen collection from confirmed cases. The articles had no language restrictions, and publications up until 5 October 2022, were included. Editorials, letters to the editor, randomized clinical trials, narrative reviews, systematic review publications, and conference proceedings were excluded.

### 2.3. Information Sources and Search Strategy

The databases of PubMed, Scopus, Web of Science, and Embase were thoroughly searched. The search terms used were: (“Monkeypox” OR “Monkey Pox”) AND “Specimen Handling” OR “Handling, Specimen” OR “Handlings, Specimen” OR “Specimen Handlings” OR “Specimen Collection” OR “Collection, Specimen” OR “Collections, Specimen” OR “Specimen Collections” OR “blood” OR “saliva” OR “skin” OR “semen” OR “genitals” OR “feces”) (Table 1). On October 5, 2022, the searches were finished, and the results were separately assessed by two distinct investigators.

### 2.4. Study Selection

Based on the electronic searches, three investigators (D.A.L.F., J.J.B. and H.M.S.-C.) assembled a database that was organized with the right management tool (EndNote), and duplicate entries were eliminated. The screening process was then carried out by two researchers (D.K.B.A. and M.J.V.-G.) using Rayyan QCRI. They separately assessed the titles and abstracts provided by the search, selected those that seemed to satisfy the inclusion criteria, and, if necessary, evaluated the complete text. If there is disagreement, the researchers will discuss their differences until a consensus is reached.

To make a choice, the authors (A.J.R.M., E.M.R. and R.S.) examined the full-text reports and considered the inclusion criteria.

### 2.5. Outcomes

The primary outcome was to analyze the detection of the monkeypox virus according to the collection site of samples from confirmed monkeypox cases.

### 2.6. Data Collection Process and Data Items

Data from the chosen papers were independently retrieved by three researchers (D.A.L.F., J.J.B., and H.M.S.-C.) and entered into a Microsoft Excel spreadsheet. The following data were extracted from the selected studies: author data, date of publication, study design, country, sex, age, risk factors, sexually transmitted infections (STIs), site of monkeypox virus-positive sample, location of skin lesions, and diagnostic test. A fourth investigator checked for duplicate articles or data in the list of publications and data extractions, and also dealt with disagreements about the inclusion of studies.

## 3. Results

### 3.1. Study Selection

**Figure 1:**
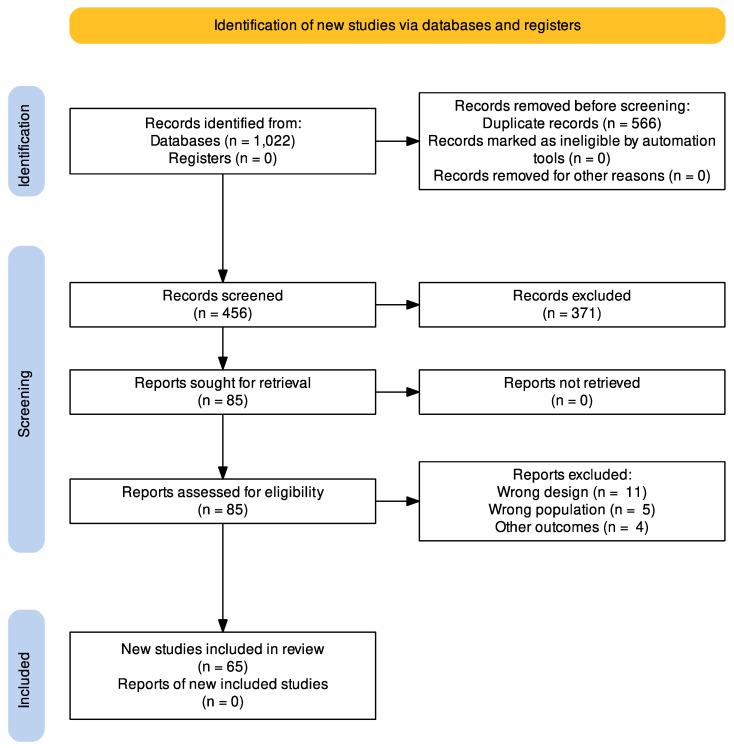
PRISMA flow chart of the studies selection process.

A total of 1022 articles were retrieved using the search strategy. The selection strategy is shown in the PRISMA flow chart (Preferred Reporting Items for Systematic Reviews and Meta-Analyses). After the removal of duplicates (*n* = 566), 456 articles were screened by the authors. After filtering the titles and reading the abstracts, 85 articles were selected for full-text reading, and 65 were considered eligible for inclusion in this systematic review (Figure 1) [18,20,21,25,26,27,28,29,30,31,32,33,34,35,36,37,38,39,40,41,42,43,44,45,46,47,48,49,50,51,52,53,54,55,56,57,58,59,60,61,62,63,64,65,66,67,68,69,70,71,72,73,74,75,76,77,78,79,80,81,82,83,84,85,86].

### 3.2. Study Characteristics

The main characteristics of the articles included in this review are summarized in Table 2 [18,20,21,25,26,27,28,29,30,31,32,33,34,35,36,37,38,39,40,41,42,43,44,45,46,47,48,49,50,51,52,53,54,55,56,57,58,59,60,61,62,63,64,65,66,67,68,69,70,71,72,73,74,75,76,77,78,79,80,81,82,83,84,85,86]. The review included 65 studies published between 1 January and 5 October 2022. The studies (*n* = 65) reported monkeypox case reports according to the specimen collection site, the number of cases, sexual behavior-oriented risk factor, history of sexually transmitted diseases, site of specimen drawn and positive for monkeypox virus by PCR, location of skin lesions, and method of diagnosis were described in detail (Table 2) [18,20,21,25,26,27,28,29,30,31,32,33,34,35,36,37,38,39,40,41,42,43,44,45,46,47,48,49,50,51,52,53,54,55,56,57,58,59,60,61,62,63,64,65,66,67,68,69,70,71,72,73,74,75,76,77,78,79,80,81,82,83,84,85,86]. A total of 4537 confirmed cases of monkeypox were reported distributed in 23 countries: Germany (*n* = 28) [21,35,47,78], India (*n* = 2) [84], Korea (*n* = 1) [70], Iran (*n* = 1) [39], Canada (*n* = 2) [55,56], Brazil (*n* = 4) [36,43,44], Spain (*n* = 2020) [32,51,72,73,74,76,81], Italy (*n* = 85) [18,20,26,42,53,54,68,71,80,83,85,86], the United Kingdom (*n* = 310) [31,33,34,59,69,77,82], Australia (*n* = 1) [60], Nigeria (*n* = 19) [30,50,75], the United States (*n* = 1217) [27,38,40,48,61,67,79], Portugal (*n* = 29) [25,62,66], France (*n* = 266) [28,45,63], Israel (*n* = 1) [29], Cameroon (*n* = 1) [37], Romania (*n* = 1) [64], Singapore (*n* = 15) [41], Central African Republic (*n* = 2) [57], Greece (*n* = 1) [49], Sweden (*n* = 1) [52], the Netherlands (*n* = 1) [46], the Czech Republic (*n* = 1) [65], and others (*n* = 528) [58]. Spain was the country with the highest number of monkeypox cases, followed by the United States and the United Kingdom.

### 3.3. Demographical Characteristics and Diagnostic Method for Monkeypox

Of the total number of cases (*n* = 4537) reported with monkeypox, 98.72% (*n* = 4479) of the cases were found to be male [18,20,21,25,26,27,28,29,30,31,32,33,34,35,36,37,38,39,40,41,42,43,44,45,46,47,48,49,50,51,52,53,54,55,56,57,58,59,60,61,62,63,64,65,66,67,68,69,70,71,72,73,74,75,76,77,78,79,80,81,82,83,84,85,86]. The mean age of the reported cases of monkeypox was 36 years [18,20,21,25,26,27,28,29,30,31,32,33,34,35,36,37,38,39,40,41,42,43,44,45,46,47,48,49,50,51,52,53,54,55,56,57,58,59,60,61,62,63,64,65,66,67,68,69,70,71,72,73,74,75,76,77,78,79,80,81,82,83,84,85,86]. Of the reported cases with monkeypox, 95.72% (*n* = 4343) [18,20,21,25,26,28,31,32,35,38,40,41,42,43,44,45,47,48,49,51,52,53,54,55,56,57,58,59,60,61,62,63,64,65,66,67,68,69,70,71,72,73,74,75,76,77,78,79,80,81,82,83,85,86] had a sexual behavior of being men who have sex with men, and 1.54% (*n* = 70) [58,61,82] of the cases had a sexual behavior of being gay or bisexual or men who have sex with men. In addition, reported cases of monkeypox with sexually transmitted infections were: 28.1% had HIV (*n* = 1274) [18,20,21,26,28,31,32,42,44,47,49,53,54,56,58,62,63,64,65,66,68,69,71,72,73,74,75,76,77,79,80,82,85,86], 2% had Syphilis (*n* = 89) [18,32,38,40,44,50,54,57,58,60,65,67,69,71,72,74,77,78,83,86], 2.1% had Gonorrhea (*n* = 94) [25,32,35,54,58,69,72,74,77,78,86], and about 1% of cases had Herpes simplex (*n* = 45) [31,32,40,58,67,69,74,77] and Chlamydia (*n* = 44) [25,32,35,58,72,74,77]. All confirmed monkeypox cases were diagnosed by reverse transcriptase polymerase chain reaction (RT-PCR) (Table 2) [18,20,21,25,26,27,28,29,30,31,32,33,34,35,36,37,38,39,40,41,42,43,44,45,46,47,48,49,50,51,52,53,54,55,56,57,58,59,60,61,62,63,64,65,66,67,68,69,70,71,72,73,74,75,76,77,78,79,80,81,82,83,84,85,86].

### 3.4. Location of Lesions, Location of Positive MPX Viral PCR Results, and the Evolution of the Disease

The most frequent locations of lesions in patients diagnosed with monkeypox (*n* = 4537) were: 42.85% on the genitalia (*n* = 1944) [18,20,29,32,35,41,42,43,44,45,46,47,48,49,50,51,52,53,54,56,58,59,60,61,62,63,64,66,67,69,70,71,72,73,74,75,76,77,78,79,80,81,83,84,85], 37.1% on the perianal region (*n* = 1683) [18,20,26,34,41,42,45,47,51,58,61,62,64,65,67,68,69,72,73,74,76,77,79,80,81,82,83], 25.21% on the face (*n* = 1144) [25,27,29,35,36,37,38,39,43,44,45,46,53,55,56,57,58,59,60,61,68,69,70,73,74,76,77,79,81,85], 19.1% on the upper and lower limbs (*n* = 867) [18,26,27,28,29,36,37,38,39,40,42,43,45,46,47,50,53,55,56,57,58,60,61,64,66,67,69,71,72,73,74,76,77,78,79,84,85], and 17.63% on the trunk (*n* = 800) [18,21,28,40,42,43,45,53,54,55,58,60,61,64,68,70,72,73,74,76,77,78,79,86] (Table 2). The diagnosis of monkeypox cases was performed by PCR [18,20,21,25,26,27,28,29,30,31,32,33,34,35,36,37,38,39,40,41,42,43,44,45,46,47,48,49,50,51,52,53,54,55,56,57,58,59,60,61,62,63,64,65,66,67,68,69,70,71,72,73,74,75,76,77,78,79,80,81,82,83,84,85,86], and the most frequent locations of samples extracted for diagnosis that tested positive for monkeypox virus (*n* = 4537) were: 91.85% from skin lesions (*n* = 4167) [18,20,21,25,26,27,29,30,31,32,33,34,35,36,37,38,39,40,41,42,43,44,45,46,47,48,49,50,51,52,53,54,55,56,57,58,59,60,61,62,63,64,65,66,67,68,69,70,71,72,73,74,75,76,77,78,79,81,82,83,84,85,86], 20.81% from oropharynx (*n* = 944) [18,21,26,28,31,35,36,45,46,47,52,58,60,62,63,64,67,68,70,71,72,73,74,83,84,85,86], 3.19% from blood (*n* = 145) [18,21,26,30,35,36,38,42,45,46,47,52,58,59,64,68,69,80,84], and 2.43% from seminal fluid (*n* = 110) [18,21,26,35,42,52,56,58,72,80] (Table 2).

The disease evolution of the cases with monkeypox was asynchronous, with no serious or severe complications [18,20,21,25,26,27,28,29,30,31,32,33,34,35,36,37,38,39,40,41,42,43,44,45,46,47,48,49,50,51,52,53,54,55,56,57,58,59,60,61,62,63,64,65,66,67,68,69,70,71,72,73,74,75,76,77,78,79,80,81,82,83,84,85,86], and most patients did not report specific treatment but simply followed symptomatic treatment [18,20,21,25,26,27,28,29,30,31,32,33,34,35,36,37,38,39,40,41,42,43,44,45,46,47,48,49,50,51,52,53,54,55,56,57,58,59,60,61,62,63,64,65,66,67,68,69,70,71,72,73,74,75,76,77,78,79,80,81,82,83,84,85,86].

## 4. Discussion

At a research center in Copenhagen, Denmark, an outbreak of a smallpox-like disease in monkeys led to the discovery and isolation of MPXV in 1958 [87]. The first human case was in 1970, when a smallpox-like disease affected a 9-month-old boy in the Democratic Republic of Congo [88].

Currently, MPXV is slowly emerging as a public health problem of international importance. Uncertainties persist regarding transmission routes; along with epidemiological data, new insights are expected from the virological assessment of the presence of MPXV in different areas of the human body [89]. Therefore, it is crucial to detect them before widespread community transmission [90]. This systematic review aims mainly to analyze the detection of MPXV according to the site of sample collection from confirmed monkeypox cases. 

A total of 4537 confirmed cases of monkeypox were reported, and 44.5% and 26.8% of the cases were distributed in Spain and the United States, respectively. It was found that 98.72% of the cases were male with an average age of 36 years, and 95.72% also had a sexual behavior of being men who have sex with men. In the evaluation of sexually transmitted infections, it was reported that 28.1% had HIV, and less than 2% had syphilis and gonorrhea. The location of the most frequent lesions was in the genital and perianal regions at 42.85% and 37.1% respectively. All cases of monkeypox were diagnosed by PCR, and the most frequent samples that tested positive for the monkeypox virus were skin lesions (91.85%) and oropharyngeal lesions (20.81%).

To address the detection of monkeypox virus DNA, according to WHO guidelines, the sample type may be (a) skin lesion material, including lesion exudate swabs, lesion ceilings, and lesion crusts; (b) oropharyngeal swabs; (c) rectal and/or genital swabs; (d) urine; (e) sperm; (f) whole blood; (g) serum; or (h) plasma [91].

The recommended specimen for diagnosis is skin lesion material, because it contains the highest concentration of the virus. In addition, the oropharyngeal swab is recommended for laboratory confirmation of cases [92]. However, because the current outbreak is still under investigation, the collection of additional specimen types for investigative purposes may be considered if permitted by the appropriate ethics review board, and there is sufficient medical and laboratory expertise for their safe collection, handling, and storage [93]. These may include serum, plasma, rectal and vaginal swabs, urine, semen, and blood.

The detection of MPXV in oropharyngeal and perioral lesions [58]. This can be a source of transmission through oral contact (kissing) and saliva exchange [59]. Similarly, the virus has been detected in anal and perianal lesions [58,76,94], generating transmission through insertive anal contact (e.g., penis or finger) or anilingus, since such exposure occurs in intimate physical contact during sexual intercourse. On the other hand, MPXV has been detected in the semen of infected men, which could plausibly transmit the infection [21]. Reda A et al. reported in their systematic review and meta-analysis study that MPXV is highly prevalent in seminal samples of MPX cases, further corroborating the role of sexual transmission of the disease [95].

DNA detection by PCR in samples can last up to 3 weeks. Samples with cycle threshold (Ct) values >35 beyond this duration have been reported for the upper respiratory tract swabs (up to 41 days and possibly 73 days) [96], saliva (up to 76 days) [52], and semen (up to 54 days) [52,58]. In addition, the proportion of replication-competent virus present has been associated with the amounts of viral DNA in clinical samples, suggesting that a higher viral load determined by PCR may indicate a greater potential for infectivity [97].

The recent outbreak of monkeypox in non-endemic regions of the world is of great concern. The current epidemiological statistics reported by WHO shows a predominance of the condition in 97.1% (40,940/42,163) of young men with a median age of 35 years (interquartile range: 29–42 years); furthermore, among cases with declared sexual orientation, 87.9% (18,549/21,099) were identified as men who have sex with men [98].

New routes of transmission of MPXV because most confirmed cases have the risk factor of sexual contact. Thus, the virus could spread rapidly between sexual partners. High viral loads of MPXV from the skin and mucosal sites, including genital and anal sites, imply that transmission is more likely to occur through direct body contact than through the respiratory route or contact with body fluids, which should help improve prevention messages sent to those most exposed to the virus [99].

The situation of this new zoonotic disease, which now appears to be emerging, warrants further study to fully understand the complex effects of this virus, which is currently affecting several continents and may have new transmission pathways, including during the current COVID-19 pandemic.

The results of this systematic review highlight findings of monkeypox virus detection according to the site of specimen collection from confirmed monkeypox cases that can assist healthcare personnel in the early recognition of cases, ensure adequate clinical monitoring and supportive interventions, and prevent further transmission through the implementation of infection control measures [100].

## 5. Limitations and Strengths

The evidence on monkeypox is constantly changing. This systematic review includes mostly case report studies and case series, which may result in heterogeneity of information. In addition, because of the current monkeypox outbreak, some high-impact studies may not have been included. In terms of strengths, the present study has a rigorous methodology, as it was conducted following the recommendations of the PRISMA guidelines. Likewise, all the processes carried out for the selection of the studies were performed independently by two or more authors.

## 6. Conclusions

Symphonic smallpox has spread rapidly throughout the world, becoming a public health problem of international importance and generating new routes of transmission with the presence of MPXV in different areas of the human body. Our findings showed that most of the cases in the current outbreak of MPX were male, with a sexual behavior of being men who have sex with men, and the most frequent lesions were in the genital and perianal regions. Finally, the most frequent locations of samples collected for diagnosis that tested positive for monkeypox virus were skin lesions, oropharynx, blood, and seminal fluid.

## Figures and Tables

**Table 1:** Bibliographic search strategy.

Base	Search Strategy
PubMed	#1 (“Monkeypox” OR “Monkey Pox”) #2 (“Specimen Handling” OR “Handling, Specimen” OR “Handlings, Specimen” OR “Specimen Handlings” OR “Specimen Collection” OR “Collection, Specimen” OR “Collections, Specimen” OR “Specimen Collections” OR “blood” OR “saliva” OR “skin” OR “semen” OR “genitals” OR “feces”) #3 = #1 AND #2
Scopus	#1 TITLE-ABS-KEY (“Monkeypox” OR “Monkey Pox”) #2 TITLE-ABS-KEY (“Specimen Handling” OR “Specimen Handlings” OR “Specimen Collection” OR “Specimen Collections” OR “blood” OR “saliva” OR “skin” OR “semen” OR “genitals” OR “feces”) #3 = #1 AND #2
Web of Science	#1 ALL = (“Monkeypox” OR “Monkey Pox”) #2 ALL = (“Specimen Handling” OR “Handling, Specimen” OR “Handlings, Specimen” OR “Specimen Handlings” OR “Specimen Collection” OR “Collection, Specimen” OR “Collections, Specimen” OR “Specimen Collections” OR “blood” OR “saliva” OR “skin” OR “semen” OR “genitals” OR “feces”) #3 = #1 AND #2
Embase	#1 ‘monkeypox’/exp OR ‘monkeypox’ OR ‘monkeypox virus’/exp OR ‘monkeypox virus’ #2 ‘specimen’ OR ‘blood’ OR ‘saliva’ OR ‘skin’ OR ‘semen’ OR ‘genitals’ OR ‘feces’ #3 = #1 AND #2

**Table 2:** Characteristics of included studies and description of case reports of monkeypox.

Authors	Year	Design	Country	Number of Cases (N)	Age (Years)	Sex (M/F)	Risk Factor	STIs	Site of Positive MPX Viral PCR	Localization of Skin Lesions	Diagnostic Method for Monkeypox
Brito Caldeira, M., et al. [25]	2022	Case report	Portugal	1	33	M	MSM	Gonorrhea and chlamydia	Skin lesions	Face and buttocks	RT-PCR
Brundu, M., et al. [26]	2022	Case report	Italy	1	35	M	MSM	HIV	Skin and anal lesions, seminal fluid, oropharynx, blood, and urine.	Abdomen, chest, back, and perianal.	RT-PCR
Costello. V., et al. [27]	2022	Case report	United States	1	28	M	None	None	Skin lesions.	Abdomen, face, neck, and hands.	RT-PCR
Davido, B., et al. [28]	2022	Case report	France	1	48	M	MSM	HIV	Throat	Peritonsillar abscess.	RT-PCR
Erez, N., et al. [29]	2019	Case report	Israel	1	38	M	None	None	Skin lesions.	Face, penis, trunk, and extremities.	RT-PCR
Eseigbe, E.E., et al. [30]	2021	Case report	Nigeria	2	20	M	None	None	Skin lesions and blood.	Skin	RT-PCR
Gedela, K., et al. [31]	2022	Case report	United Kingdom	2	40	M	MSM	Herpes simplex (*n* = 2), HIV (*n* = 1)	Perianal, rectum, nose, and throat ulcers.	Skin	RT-PCR
					30						
Gomez-Garberi, M., et al. [32]	2022	Observational study	Spain	14	Median: 40 (20–56)	M	MSM (*n* = 10) and Heterosexuals (*n* = 2)	HIV (*n* = 8), Chlamydia (*n* = 2), Herpes type 2 (*n* = 1), Syphilis (*n* = 1), Mycoplasma genitalium (*n* = 1), and Gonococcus (*n* = 1)	Skin lesions (vesicles, pustules, and crusts)	Skin lesion, genital area, penis, and scrotum.	RT-PCR
Hobson, G., et al. [33]	2022	Case report	United Kingdom	3	NR	M	None	None	Skin lesions	Skin	RT-PCR
Hofer, U., [34]	2022	Case series	United Kingdom	7	NR	NR	None	None	Nose and throat	Skin	RT-PCR
Hornuss, D., et al. [35]	2022	Case series	Germany	4	Range (29–53)	M	MSM	Chlamydia (*n* = 1), Gonorrhoea (*n* = 1), and Mycoplasma (*n* = 1)	Typical lesions (vesicle, encrustation, erosion, ulceration), forearms/palms, pharyngeal/anal mucosa, blood, urine, and seminal fluid.	Mons pubis (*n* = 1), Perioral (*n* = 1), Perianal (*n* = 2), Nose (*n* = 1), and Genital (*n* = 2)	RT-PCR
Carvalho, L.B., et al. [36]	2022	Case report	Brazil	1	20	F	None	None	Blood, skin lesions and oropharyngeal lesions.	Hands, left thigh, and face.	RT-PCR
Jarman, E.L., et al. [37]	2022	Case report	Cameroon	1	14	M	None	None	Suprapubic injury and hand	Face, head, chest, arms, and legs.	RT-PCR
Karan, A., et al. [38]	2022	Case report	United States	1	20	M	MSM	Syphilis	Plasma and skin lesions.	Skin lesions, hands, lip, and back.	RT-PCR
Karbalaei, M., et al. [39]	2022	Case report	Iran	1	34	F	None	None	Skin lesions	Skin lesions and hands.	RT-PCR
Khan, S., et al. [40]	2022	Case report	United States	1	31	M	MSM	Herpes simplex, varicella-zoster, and syphilis.	Skin lesions	Hands, feet, and trunk.	RT-PCR
Koh, X.Q., et al. [41]	2022	Case series	Singapore	15	Median: 38.4 (25–54)	M	MSM	NR	Skin lesions	Skin lesions (*n* = 11), inguinal and anogenital regions (*n* = 5), and abdomen (*n* = 1).	RT-PCR
Lapa, D., et al. [42]	2022	Case report	Italy	1	39	M	MSM	HIV	Plasma, semen, rash, or skin lesion.	Anus, head, thorax, legs, arms, hand, and penis.	RT-PCR
Lima, E.L., et al. [43]	2022	Case report	Brazil	1	41	M	MSM	None	Skin lesions	Face, periumbilical region, back, upper extremities, trunk, and genitals.	RT-PCR
Lopes, P.S., et al. [44]	2022	Case report	Brazil	2	28	M	MSM	Syphilis (*n* = 2)	Skin lesions	Lip (*n* = 2) and penis (*n* = 1)	RT-PCR
Mailhe, M., et al. [45]	2022	Observational study	France	264	Median: 35 (30-41)	M (*n* = 262) F (*n* = 1) Trans (*n* = 1)	MSM (*n* = 245)	HIV (*n* = 73) and history of STI (*n* = 209)	Skin (*n* = 252), oropharynx (*n* = 150), and blood (*n* = 8).	Genital area (*n* = 135), limbs (*n* = 121), torso (*n* = 105), perianal area (*n* = 100), face (*n* = 88), and palmoplantar area (*n* = 36).	RT-PCR
Tutu van Furth, A.M., et al. [46]	2022	Case report	Netherlands	1	10	M	None	None	Blood, throat, anal region, and skin vesicles.	Face, ear, jaw, forearms, shoulder, thighs, and back.	RT-PCR
Nörz, D., et al. [47]	2022	Case reports	Germany	16	Range (20–40)	M	MSM	HIV (*n* = 2)	Skin lesions (*n* = 16), blood (*n* = 4) and oropharynx (*n* = 3)	Anal/perianal (*n* = 3), genital/perigenital (*n* = 8), oral (*n* = 2), face (*n* = 1), oral (*n* = 1), and arm (*n* = 2).	RT-PCR
Ortiz-Martínez, Y., et al. [48]	2022	Case report	United States	1	36	M	MSM	None	Skin lesions	Penis, neck, thigh, and nipple	RT-PCR
Paparizos, V., et al. [49]	2022	Case report	Greece	1	59	M	MSM	HIV	Skin lesions	Pubic area and penis.	RT-PCR
Pembi, E., et al. [50]	2022	Case report	Nigeria	1	30	M	None	Syphilis	Skin lesions	Forehead, groin, genitals, chest, back, feet, and hands.	RT-PCR
Pérez-Martín, Ó.G., et al. [51]	2022	Case report	Spain	1	40	M	MSM	None	Skin lesions	Perineum, penis, and testicles.	RT-PCR
Pettke, A., et al. [52]	2022	Case report	Sweden	1	NR	M	MSM	None	Genital lesions, blood, urine, saliva, nasopharynx, and semen.	Genital skin lesions.	RT-PCR
Pipitò, L., et al. [53]	2022	Case report	Italy	1	45	M	MSM	HIV	Skin lesions	Face, neck, genitalia, extremities, and trunk.	RT-PCR
Quattri, E., et al. [54]	2022	Case report	Italy	2	35 and 39	M	MSM	HIV (*n* = 2), Syphilis (*n* = 2), Gonorrhea (*n* = 1)	Skin lesions	Lesión cutánea (*n* = 2), prepucio (*n* = 1), pene (*n* = 1), and tronco (*n* = 1).	RT-PCR
Sukhdeo, S.S., et al. [55]	2022	Case report	Canada	1	33	M	MSM	None	Pustules of each arm and serum.	Face, extremities, torso, forearm, and wrist.	RT-PCR
Tan, D.H.S., et al. [56]	2022	Case report	Canada	1	40	M	MSM	HIV	Skin lesions, saliva, and semen.	Cutaneous, genital, labial, chest, and arm lesions.	RT-PCR
Berthet, N., et al. [57]	2011	Case report	Central African Republic	2	14 and 15	M	None	Syphilis (*n* = 1)	Skin lesions	Skin, face, torso, and extremities.	RT-PCR
Thornhill, J.P., et al. [58]	2022	Case report	Multicountry (*n* = 16)	528	Median: 38 (18–68)	M (*n* = 527) Trans (*n* = 1)	Homosexual (*n* = 509) Bisexual (*n* = 10)	HIV (*n* = 218) Gonorrhea (*n* = 32/377), Chlamydia (*n* = 20/377), Syphilis (*n* = 33/377), Herpes simplex (*n* = 3/377), and Lymphogranuloma venereum (*n* = 2/377)	Skin or anogenital lesion (*n* = 512), Nose or throat swab (*n* = 138), Blood (*n* = 35), Urine (*n* = 14), and Semen (*n* = 29)	Anogenital area (*n* = 383), face (*n* = 134), trunk or limbs (*n* = 292), palms or soles (*n* = 51), and mu-cosal lesions present (*n* = 217).	RT-PCR
Antinori, A., et al. [18]	2022	Case reports	Italy	4	Median: 30	M	MSM	Hepatitis C (*n* = 1), syphilis (*n* = 3), hepatitis B (*n* = 1), Hepatitis A (*n* = 1), HIV (*n* = 2).	Serum (*n* = 1), Plasma (*n* = 1), Genital or rectal lesions (*n* = 4), Nasopharyngeal swab (*n* = 3), Skin lesions (*n* = 3), Seminal fluid (*n* = 3), Scab (*n* = 2), Faeces (*n* = 2), and Saliva (*n* = 1)	Genital (*n* = 3), thorax (*n* = 2), Anal (*n* = 2), arms (*n* = 2)	RT-PCR
Heskin, J., et al. [59]	2022	Case reports	United Kingdom	2	NR	M	MSM	None	Serum (*n* = 2), genital lesions (*n* = 2), and urine (*n* = 2).	Genital (*n* = 2), pubic and tongue (*n* = 2), oral and buccal mucous membranes (*n* = 2)	RT-PCR
Hammerschlag, Y., et al. [60]	2022	Case report	Australia	1	30	M	MSM	Syphilis	skin lesions and nasal throat	Penis, trunk, face, extremities, hand, calf, nasal throat.	RT-PCR
Minhaj, F.S., et al. [61]	2022	Case reports	United States	17	Median 40 (28–61)	M	GBMSM (*n* = 16)	NR	Skin lesions	Arm (*n* = 9), Trunk (*n* = 9), Leg (*n* = 8), Face (*n* = 7), Hand (*n* = 6), Perianal (*n* = 6), Oral (*n* = 5), Neck (*n* = 5), Genital (penis or vagina) (*n* = 4), Feet (*n* = 4).	RT-PCR
Perez Duque, M., et al. [62]	2022	Case reports	Portugal	27	Median: 33 (22–51)	M	MSM (18/19), MSW (1/19)	HIV (*n* = 14)	Lesions on the palms of the hands, genital area, and/or oral mucosa.	Anus (*n* = 14) and genitalia (*n* = 12)	RT-PCR
Vallée, A., et al. [63]	2022	Case report	France	1	NR	M	MSM	HIV	Pharyngeal area	Genitalia	RT-PCR
Oprea, C., et al. [64]	2022	Case report	Romania	1	26	M	MSM	HIV	Skin, nasopharyngeal, urine, and blood lesions.	Anogenital, buttocks, neck, trunk, upper and lower limbs, and sole of one foot.	RT-PCR
Bížová, B., et al. [65]	2022	Case report	Czech Republic	1	34	M	MSM	Syphilis and HIV	Perianal	The perianal and left side of the body.	RT-PCR
Patrocinio-Jesus, R., et al. [66]	2022	Case report	Portugal	1	31	M	MSM	HIV	Genitals and hands	Genitals and hands	RT-PCR
Basgoz, S.N., et al. [67]	2022	Case report	United States	1	31	M	MSM	Syphilis, herpes simplex	Skin lesions, throat, and serum	Perianal, penis, arms, and legs.	RT-PCR
Mileto, D., et al. [68]	2022	Case report	Italy	1	33	M	MSM	HIV	Oropharynx, anus, perianal ulcerated lesion, a foot vesicle, and plasma.	Perianal, face, both elbows, trunk, buttock, and right foot.	RT-PCR
Girometti, N., et al. [69]	2022	Cohort study	United Kingdom	54	Median: 41 (34–45)	M	MSM	HIV (*n* = 13) syphilis (*n* = 14), herpes simplex (*n* = 24) and gonorrhea (*n* = 13)	Blood, urine, and skin lesions.	Skin (*n* = 54), genitalia (*n* = 33), perianal (*n* = 24), upper and lower extremities (*n* = 27), facial (*n* = 11), oropharyngeal (*n* = 4) and torso (*n* = 14).	RT-PCR
Noe, S., et al. [21]	2022	Case report	Germany	2	26	M	MSM	HIV (*n* = 1)	Blood, semen, throat, and skin lesions.	Tonsils, trunk, limbs, and head.	RT-PCR
					32						
Jang, Y.R., et al. [70]	2022	Case report	Korea	1	34	M	MSM	None	Penile, oropharyngeal, and nasopharyngeal.	Penis, oropharynx, nasopharynx, face, abdomen, and trunk.	RT-PCR
Maronese, C.A., et al. [71]	2022	Case report	Italy	1	44	M	MSM	Hepatitis C, HIV, syphilis	Pharyngeal and skin lesions.	Penis, scrotum, and extremities.	RT-PCR
Peiró-Mestres, A., et al. [72]	2022	Case report	Spain	12	Range (30–50)	M	MSM	HIV (*n* = 4), Syphilis (*n* = 2) Chlamydia (*n* = 1) y gonorrhea (*n* = 1).	Saliva (*n* = 12), rectal (*n* = 11), nasopharyngeal (*n* = 10), semen (*n* = 7), urine (*n* = 9), and feces (*n* = 8).	Arm (*n* = 1), trunk (*n* = 3), genital area (*n* = 5), anal area (*n* = 6), chest (*n* = 2), legs (*n* = 1), pectoral (*n* = 1), fingers (*n* = 1), and hand (*n* = 1).	RT-PCR
Iñigo Martínez, J., et al. [73]	2022	Case report	Spain	508	Median: 35 (18–67)	M (*n* = 503) F (*n* = 5)	MSM (*n* = 397)	HIV (*n* = 225)	Skin lesions, urine, pharyngeal exudates, and mucosal exudates.	Anogenital and/or perineal area (*n* = 359), legs and/or arms (*n* = 222), face (*n* = 177), chest and/or abdomen (*n* = 159), back (*n* = 132), palms and/or plants (*n* = 124).	RT-PCR
Tarín-Vicente, E.J., et al. [74]	2022	Cohort study	Spain	181	Median: 37 (31–42)	M (*n* = 175) F (*n* = 6)	MSM (*n* = 166) MSW (*n* = 15)	HIV (*n* = 72), Chlamydia (*n* = 10), gonorrhea (*n* = 6), herpes simplex virus (*n* = 2), and syphilis (*n* = 13).	Skin (*n* = 178/180), throat (*n* = 82/117), and anal (*n* = 43/55) lesions.	Genital (*n* = 100), Perianal (*n* = 66), Oral ulcer (*n* = 45), Perioral (*n* = 51), Hands and feet (*n* = 108), Trunk and extremities (*n* = 104)	RT-PCR
Ogoina, D., et al. [75]	2022	Cross-sectional study	Nigeria	16	Median: 28 (22–43)	M (*n* = 12) F (*n* = 4)	MSW	HIV (*n* = 3)	Skin lesions	Genital (*n* = 13)	RT-PCR
Orviz, E., et al. [76]	2022	Observational study	Spain	48	Median: 35 (29–44)	M	MSM (*n* = 42)	HIV (*n* = 19)	Skin lesions	Vesicular-umbilicated skin lesions location (*n* = 45), Genitals (*n* = 26), Upper extremities (*n* = 20), Perianal (*n* = 17), Trunk (*n* = 16), Facial (*n* = 12), Periorally (*n* = 9), Lower extremities (*n* = 10), and Palms and soles (*n* = 2)	RT-PCR
Patel, A., et al. [77]	2022	Case report	United Kingdom	197	Median: 38 (32–42)	M	MSM	HIV (*n* = 70), Chlamydia (*n* = 11), gonor-rhea (*n* = 34), herpes simplex virus (*n* = 11), and syphilis (*n* = 6).	Skin lesions	Face (*n* = 71), Trunk (*n* = 70), Arms/legs (*n* = 74), Hands/feet (*n* = 56), Genitals (*n* = 111), Anus or perianal area (*n* = 82), and Oropharyngeal (*n* = 27)	RT-PCR
Pfäfflin, F., et al. [78]	2022	Case report	Germany	6	Range (21–50)	M	MSM	Syphilis (*n* = 1), gonorrhea (*n* = 3), myco-plasma homi-nis (*n* = 1)	Skin blister fluid	Limbs (*n* = 3), arm (*n* = 2), trunk (*n* = 2), genital (*n* = 1), head (*n* = 1), neck (*n* = 1)	RT-PCR
Philpott, D., et al. [79]	2022	Case report	United States	1195	Median: 35 (30–41)	M (*n* = 1178) F (*n* = 5)	MSM	HIV (*n* = 490)	Skin rash	Genitals (*n* = 333), Arms (*n* = 284), Face (*n* = 276), Legs(*n* = 265), Perianal (*n* = 225), Mouth, lips, or oral mucosa (*n* = 179), Palms of hands (*n* = 157), Trunk (*n* = 156), Neck (*n* = 130), Head (*n* = 97), and Soles of feet (*n* = 77)	RT-PCR
Raccagni, A.R., et al. [80]	2022	Case report	Italy	36	Median: 41.5 (31.25–35.5)	M	MSM	HIV (*n* = 15)	Hyssop (*n* = 36), Seminal fluids (*n* = 22), Urines (*n* = 8), and Serum/Plasma (*n* = 24)	Genital (*n* = 13), Rectal (*n* = 18), cutaneous (*n* = 20)	RT-PCR
Rodríguez, B.S., et al. [81]	2022	Case report	Spain	1256	Median: 37	M (*n* = 1242) F (*n* = 14)	MSM	NR	Skin lesions	Report of some cases (*n* = 530): Anogenital (*n* = 355), other than anogenital or oro/peribuccal (*n* = 293)	RT-PCR
Vusirikala, A., et al. [82]	2022	Case report	United Kingdom	45	Median: 37	M	GBMSM (*n* = 44)	HIV (*n* = 11)	Skin lesions	Skin lesions	RT-PCR
Moschese, D., et al. [20]	2022	Case report	Italy	33	Median: 38 (34–43)	M	MSM	HIV (*n* = 17)	Anus (*n* = 13/18) and urethra (*n* = 11/15).	Cutaneous (*n* = 33), perianal (*n* = 7) and glans (*n* = 3) lesions.	RT-PCR
Ramoni, S., et al. [83]	2022	Case report	Italy	2	24	M	MSM	Syphilis	Skin lesions	Pubic area	RT-PCR
					38	M	MSM	Syphilis	Skin and pharyngeal lesions	Penis, perianal region, and forehead.	
Yadav, P.D., et al. [84]	2022	Case report	India	2	35	M	None	None	Skin lesions, oropharyngeal, nasopharyngeal, blood, serum, and urine.	Oral cavity, lips, genitalia, and navel.	RT-PCR
					31	M	None	None	Skin lesions, oropharyngeal, nasopharyngeal, and urine.	Genitals, hands, face, back, neck, and forearm.	
Turco, M., et al. [85]	2022	Case report	Italy	1	46	M	MSM	None	Skin lesions and the oropharynx.	Face, hands, and penis.	RT-PCR
Pisano, L., et al. [86]	2022	Case report	Italy	2	45	M	MSM	HIV, syphilis, gonorrhoea.	Oral lesions and the oropharynx.	Oral mucosa and trunk	RT-PCR
					69	M	MSM	HIV, syphilis, hepatitis C.	Oral lesions, oropharynx, and nipple.	Oral mucosa and nipple	

MSM:  men who have sex with men; MSW: men who have sex with women; GBMSM: gay or bisexual or other men who have sex with men; STI: sexually transmitted infection; HIV: human immunodeficiency virus; RT-PCR: Polymerase chain reaction with reverse transcriptase; M/F: Male/Female; NR:  No report.
